# How embarrassing! The behavioral and neural correlates of processing social norm violations

**DOI:** 10.1371/journal.pone.0176326

**Published:** 2017-04-25

**Authors:** Janna Marie Bas-Hoogendam, Henk van Steenbergen, Tanja Kreuk, Nic J. A. van der Wee, P. Michiel Westenberg

**Affiliations:** 1 Institute of Psychology, Leiden University, Leiden, The Netherlands; 2 Department of Psychiatry, Leiden University Medical Center, Leiden, The Netherlands; 3 Leiden Institute for Brain and Cognition, Leiden, The Netherlands; Mälardalen University, SWEDEN

## Abstract

Social norms are important for human social interactions, and violations of these norms are evaluated partly on the intention of the actor. Here, we describe the revised Social Norm Processing Task (SNPT-R), a paradigm enabling the study of behavioral and neural responses to intended and unintended social norm violations among both adults and adolescents. We investigated how participants (adolescents and adults, *n* = 87) rate intentional and unintentional social norm violations with respect to inappropriateness and embarrassment, and we examined the brain activation patterns underlying the processing of these transgressions in an independent sample of 21 adults using functional Magnetic Resonance Imaging (fMRI). We hypothesized to find activation within the medial prefrontal cortex, temporo-parietal cortex and orbitofrontal cortex in response to both intentional and unintentional social norm violations, with more pronounced activation for the intentional social norm violations in these regions and in the amygdala. Participants’ ratings confirmed the hypothesis that the three types of stories are evaluated differently with respect to intentionality: intentional social norm violations were rated as the most inappropriate and most embarrassing. Furthermore, fMRI results showed that reading stories on intentional and unintentional social norm violations evoked activation within the frontal pole, the paracingulate gyrus and the superior frontal gyrus. In addition, processing unintentional social norm violations was associated with activation in, among others, the orbitofrontal cortex, middle frontal gyrus and superior parietal lobule, while reading intentional social norm violations was related to activation in the left amygdala. These regions have been previously implicated in thinking about one’s self, thinking about others and moral reasoning. Together, these findings indicate that the SNPT-R could serve as a useful paradigm for examining social norm processing, both at the behavioral and the neural level.

## Introduction

In the present work, we describe the revised Social Norm Processing Task (SNPT-R), a paradigm enabling the study of behavioral and neural responses to intended and unintended social norm violations among both adults and adolescents. More specifically, we investigated how participants rate intentional and unintentional social norm violations with respect to inappropriateness and embarrassment, and we examined the brain activation patterns underlying the processing of these transgressions.

Social norms are crucial in creating and maintaining social relationships, because they specify what is acceptable in a certain social group [1,2]. Transgressions of these norms induce self-conscious emotions like embarrassment and guilt [3–5]. These emotions are prosocial, because they lead to action tendencies which are important to restore the social order [6–8].

Several studies have investigated the behavioral and neural responses to violations of norms and the associated prosocial emotions, for example while focusing on making moral judgments [9–11], the emergence of human social values [12], the effect of the presence or absence of an audience on processing moral and social transgressions [13], and the experience of self-conscious moral emotions like shame and guilt [14–16]. While these paradigms investigated several aspects of norm processing, the focus of the Social Norm Processing Task (SNPT), originally developed and described by Berthoz et al. [17,18] and used in a subsequent study [19], is on the effect of intention on the judgment of social norm violations. The concept of ‘intentionality’ has been extensively studied [20–22] and the effect of the actor’s intention on the evaluation of an action has been shown previously (cf. [9–11]). For example, intentional harmful acts were judged worse [9] and more ‘wrong’ [10] than accidental harmful acts.

In the SNPT, participants read and evaluate stories describing neutral social situations and situations in which social norms are intentionally or unintentionally transgressed [17]. Social norms, in this task, are widely shared beliefs on appropriate behavior in a social situation, i.e. in a situation where others are present. It should, however, be noted that several other definitions of ‘social norms’ exist, for example in the context of economic decision games [23–27]. Furthermore, there is a debate about how social norms are different from moral norms and decency norms, a discussion which is outside the scope of this paper [2,28–30].

Results on the SNPT [17] revealed that participants evaluated the stories differently with respect to inappropriateness and embarrassment: healthy male participants (*n* = 12) rated intentional social norm violations as more inappropriate when compared to unintentional norm violations, while they considered the unintentional norm violations as the most embarrassing [17,18]. These findings indicate that the evaluation of social norm violations is determined to a great extent by the intention of the actor [18], given the fact that the consequences of the intentional and the unintentional social norm violations are in general the same [17]. Furthermore, neuroimaging data on the SNPT indicated that reading stories describing intentional and unintentional social norm violations evoked activation within the medial and superior prefrontal cortex, the left temporo-parietal junction and left orbito-frontal cortex, while the intentional condition (compared to unintentional condition) was associated with stronger activation within the medial and superior frontal cortex, anterior cingulate gyrus, parietal regions including the precuneus, left superior occipital gyrus and, as shown in a re-analysis of the data [18], the left amygdala [18].

In addition to the study by Berthoz et al. on healthy male participants [17], the SNPT was used in an imaging study on social anxiety disorder (SAD) [19]. Patients with SAD are characterized by an intense fear of negative evaluation [31], which was reflected by aberrant behavioral and neural responses to the SNPT. At the behavioral level, SAD patients (*n* = 16) reported higher levels of inappropriateness and embarrassment across all conditions (intentional, unintentional and neutral), when compared to healthy control participants (*n* = 16). Furthermore, increased activation in the medial prefrontal cortex in response to unintentional norm violations was present in SAD [19], suggesting altered self-referential processing. These findings indicate that the SNPT is a useful paradigm to investigate the neurobehavioral correlates of social anxiety, but we suggest, in line with Berthoz and colleagues [17], that the SNPT can also be utilized in future research on the vulnerability for other psychiatric and neurological conditions in which social behavior is typically affected.

However, previous work on the SNPT [17,19] has several limitations, hindering its future use. Both studies had small sample sizes (*n* = 12 [17] and *n* = 16 healthy participants [19]), and included only adult participants, while Berthoz and colleagues [17] examined solely males. Furthermore, given the focus of these studies on the neural correlates of social norm processing, behavioral responses were not described in detail. In addition, different versions of the SNPT were used: while the SNPT employed by Blair and colleagues [19] only comprised impersonal stories (i.e. the story protagonist is a character like ‘Joanna’), Berthoz et al. [17,18] used a combination of personal and impersonal stories (i.e. the story protagonist is ‘you’ or the story protagonist is a character like ‘Joanna’, respectively), as well as ‘nonsense’ stories composed of unrelated words, which were not further analyzed. Furthermore, the imaging parameters of the paradigms vary to a great extent: the paradigm by Berthoz and colleagues [17,18] has a duration of more than 50 minutes, while the task used by Blair et al. lasts around 15 minutes [19]. Finally, the stories of these SNPT-versions are not publicly available. Taken together, these differences make it hard to compare the results of these studies and to obtain a clear picture of social norm processing in healthy participants, which could serve as a reference for future studies in patients.

Here, we describe, building upon the work of Berthoz et al. [17,18] and Blair et al. [19], an adapted version of the SNPT: the revised Social Norm Processing Task (SNPT-R). In line with previous versions of the SNPT, the SNPT-R contains stories describing neutral social situations, stories on unintentional social norm violations, and stories depicting intentional social norm violations. However, in contrast to earlier versions of the SNPT [17–19], the SNPT-R uses only personal stories in order to maximize personal involvement of the participants while reading the stories (cf. [13]). In line with this, we developed four age- and gender specific versions of SNPT-R, making the paradigm appropriate for participants of different ages. Other changes relative to previous versions of the SNPT involve a shortening of the duration of the paradigm relative to the paradigm by Berthoz et al. [17], mainly by omitting the ‘nonsense’ stories, and improvements in the fMRI design like the use of a jittered presentation of a fixation cross between the stories.

Main aim of the present study was to validate the SNPT-R, by replicating the findings of previous versions of the SNPT. First, we examined the behavioral ratings of inappropriateness and embarrassment for the three types of stories in a sample of adolescents and adults (*n* = 87). We hypothesized to find an effect of intention on the evaluation of the stories, both on the ratings of inappropriateness and embarrassment, as reported previously [17]. Secondly, we investigated neural responses to the stories using fMRI, in an independent sample of 21 adults, aiming to replicate the results described by Berthoz et al. [17,18]. More specifically, we expected to find activation within the medial prefrontal cortex, temporo-parietal cortex and orbitofrontal cortex in response to both intentional and unintentional social norm violations, with more pronounced activation for the intentional social norm violations in these regions [17]. Furthermore, we hypothesized that intentional social norm violations would be associated with left amygdala activation as reported by [18].

The present study extends previous work on the SNPT in two ways. First, we use a larger sample of participants, including both genders and with a broader age range. Secondly, by publishing the stories used in the SNPT-R (S1 Table and osf.io/m8r76 [32]), as well as the code for stimulus presentation (available on osf.io/m8r76 [32]) and the data acquired in the present study (osf.io/m8r76 [32] and http://neurovault.org/collections/QCZKVNWZ/), we aim to encourage the use of the SNPT-R in future studies.

## Materials and methods

### Participants

One hundred eight participants were included in the study, divided into two independent samples. Sample size was determined by availability of subjects and resources. The first sample (from now on referred to as ‘behavioral sample’) consisted of adolescents and adults who performed the SNPT-R on a laptop or personal computer, while the second sample was comprised of adults who were scanned using fMRI while reading the stories (‘imaging sample’). All participants were required to have Dutch as their first language, to be in good health and to be free of past and present psychopathology as assessed by a self-report questionnaire. General contraindications for undergoing an MRI scan and left-handedness, evaluated by a self-report questionnaire, were exclusion criteria for the imaging sample.

Ninety-four participants signed up for the behavioral experiment; four participants were excluded from participation because they did not meet the selection criteria (*n* = 3: present medication use; *n* = 1: present phyisical disorder). Furthermore, data from three participants were excluded from analysis because they performed a version of the SNPT-R that did not match their age, leaving a total of 87 participants in the behavioral sample.

Twenty-three participants were screened for participation in the imaging study; one participant was excluded due to past psychopathology, one MRI session was aborted due to participant claustrophobia, resulting in a sample of 21 participants. A neuroradiologist examined all structural MRI scans; no clinically relevant abnormalities were present in any of the participants included in the imaging sample.

All participants (and in case of minors below 18 years of age, both parents) signed informed consent prior to participation. The study was approved by the Psychology Research Ethics Committee of Leiden University (behavioral sample; study numbers 2282269557 and 8070826266) and the Medical Ethical Committee of the Leiden University Medical Center (imaging sample; protocol number P12.061). Participants were recruited via flyers, in-class announcements and by word of mouth and tested between July 2013 and December 2015 (imaging sample: July—August 2013; behavioral sample adults: November—December 2014; behavioral sample adolescents: June 2015—December 2015). After performing the experiment, participants were debriefed about the aim of the study and received a compensation for partaking in the experiment (imaging sample: monetary reward; behavioral sample adults: study credits; behavioral sample adolescents: chocolate bar).

Sample-characteristics are summarized in Table 1. Participants of the behavioral sample were divided into four groups (group 1: boys < 18 years of age; group 2: girls < 18 years of age; group 3: men ≥ 18 years of age; group 4: women ≥ 18 years of age), based on the four age- and gender specific versions of the SNPT-R. As a consequence, groups differed significantly with respect to age (oneway ANOVA: F(3,86) = 59.0, p < 0.001): boys and girls did not differ in age (independent-samples t-test: t(27) = -0.38, *ns*), but the men were significantly older when compared to the women (t(35.8) = 3.1, p = 0.004). In the imaging sample, there were no age differences between men and women (t(19) = 0.41, *ns*).

**Table 1:** Characteristics participants.

**Behavioral sample**	**Boys (*n* = 13)**	**Girls (*n* = 16)**	**Men (*n* = 29)**	**Women (*n* = 29)**
Age in years	14.0 ± 1.2 (12.7–16.5)	14.2 ± 1.4 (12.5–17.0)	21.1 ± 3.1 (18.5–32.6)	19.2 ± 1.2 (18.1–24.1)
**Imaging sample**			**Men (*n* = 6)**	**Women (*n* = 15)**
Age in years			25.8 ± 9.3 (18.7–44.1)	24.0 ± 9.7 (18.1–57.1)

Values are expressed as mean ± SD (range).

### Social Norm Processing Task (SNPT-R)

**Fig 1:**
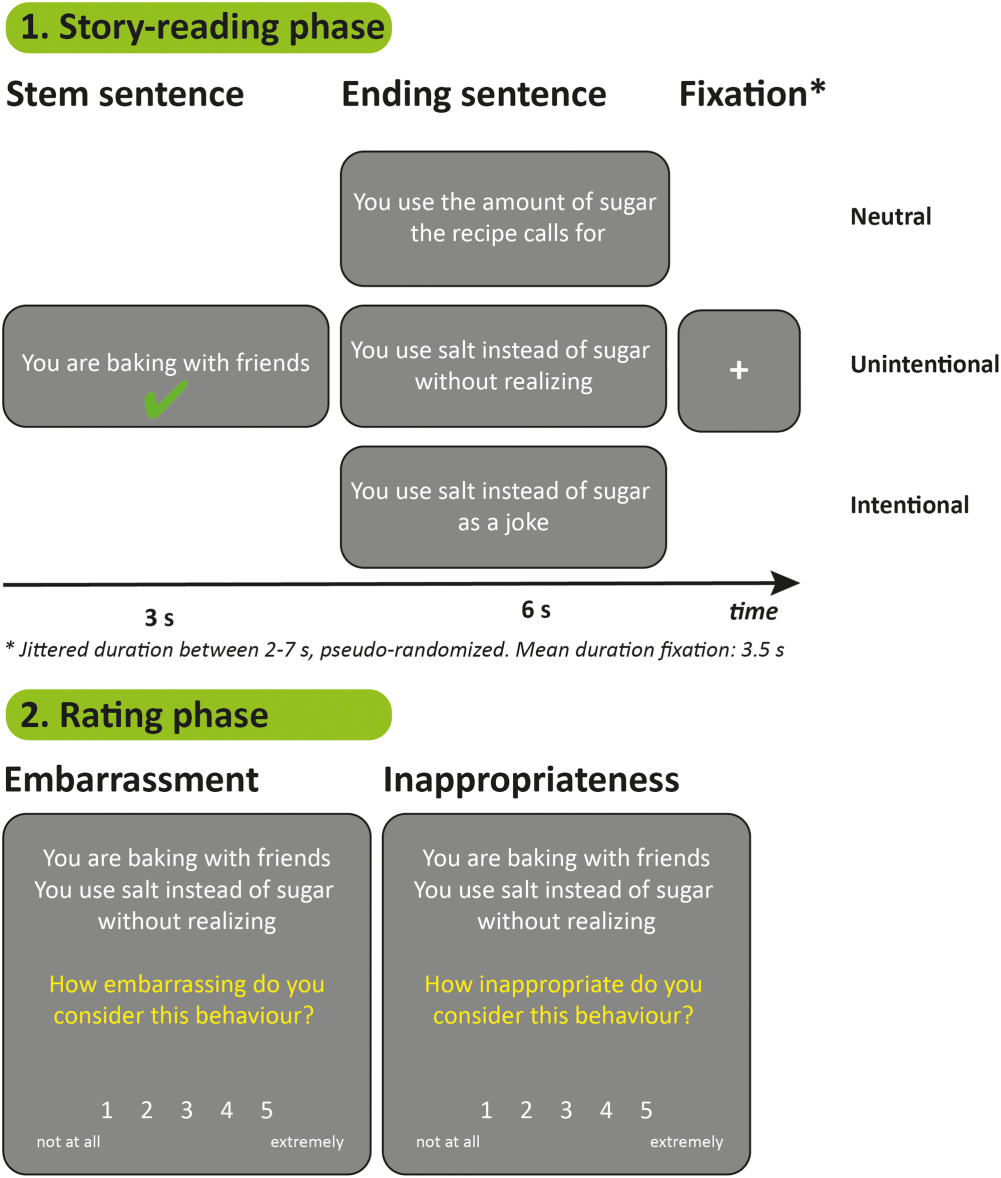
Overview of the revised Social Norm Processing Task (SNPT-R). During the story-reading phase (1), participants read stories consisting of a stem sentence and an ending sentence, describing either a neutral social situation, a situation in which a social norm was unintentionally transgressed or situation in which a social norm was violated intentionally. Participants were instructed to imagine themselves in the situation described. In the rating phase (2), participants rated all stories on embarrassment and inappropriateness.

Participants performed the revised Social Norm Processing Task (SNPT-R), an adaptation of the Social Norm Processing Task described by [17–19]. The SNPT-R consists of two phases: a story-reading phase and a rating phase (Fig 1).

In the story-reading phase, participants read short stories written in second person. Each story consisted of two sentences, a stem sentence (duration: 3 s) and an ending sentence (duration: 6 s). The stories described either a situation in which no social norm was transgressed (“neutral condition”), a situation in which a social norm was unintentionally transgressed (“unintentional condition”) or a situation in which a social norm was intentionally transgressed (“intentional condition”). It is important to note that the unintentional (“You are baking with friends. You use salt instead of sugar without realizing.”) and intentional (“You are baking with friends. You use salt instead of sugar as a joke.”) condition differ only in the intention of the actor, while we aimed to keep the actual outcome of the action (for example, a distasteful cake) in general the same (although the outcome of some intentional stories could be considered to be more severe in comparison to the outcome of the matching unintentional story, inherent to the verb used to describe intentionality; we refer the reader to S1 Text for a sensitivity analysis).

The stories in the SNPT-R were developed in collaboration with Karina S. Blair, author of previous work on the SNPT [19]. All stories described everyday social situations, in which the protagonist was accompanied by at least one other person, and the stories outlined relative innocuous violations of conventional social norms, in which no severe harm was done to others. The stories were heterogeneous with respect to the context (for example, in the presence of one friend or in public space like an airport) and the nature of the social norm transgression (for example, breaking decency rules versus hurting somebody), in order to increase the external validity of the paradigm. Stories were developed to be suitable for a broad audience and age-range (for children from age 8). However, given the fact that the stories of the SNPT-R were all personal (written in ‘you’ form) in order to maximize personal involvement of participants, some small changes were necessary in stories describing age- or genderspecific elements. Therefore, four age- and gender specific versions of the task were developed: for boys < 18 years of age (version 1), girls < 18 years of age (version 2), men ≥ 18 years of age (version 3) and women ≥ 18 years of age (version 4). For example, the school environment (< 18 years) was replaced for a work environment (≥ 18 years of age), and ‘bikini bottoms’ (females) for ‘swimming trunks’ (males). However, these changes were only minimal (see S1 Table and osf.io/m8r76 for a full list of stories included in the SNPT-R [32]).

In line with the SNPT described by Blair et al. [19], twenty-six stem sentences were developed, with three different types of ending. Therefore, the SNPT-R consisted of 78 short stories in total. These stories were presented in a pseudo-random order using E-Prime software (version 2.0.10, Psychology Software Tools; script available at osf.io/m8r76 [32]), separated by a fixation cross (jittered duration between 2–7 s, determined using Optseq software (https://surfer.nmr.mgh.harvard.edu/optseq/), mean duration fixation: 3.5 s) and divided into two consecutive blocks of 39 stories (duration each block: 8 min 44 s). Participants were instructed to imagine themselves in the social situations described and to press a button with their right index finger after reading the stem sentence of each story. A button press within 3 s resulted in visual feedback to the participant (a green checkmark presented beneath the sentence). This element was added to the paradigm in order to be able to check whether participants engaged with the task. Prior to the start of the experiment, all participants were familiarized with the story-reading phase by performing a short version of the task (using 5 stories).

In the (unannounced) rating phase of the task (Fig 1), participants read all stories again and were asked to rate them on a 5-point Likert scale on embarrassment (ranging from 1, not embarrassing at all, to 5, extremely embarrassing) and inappropriateness (ranging from 1, not inappropriate at all, to 5, extremely inappropriate), similar as in the SNPT described by Blair and colleagues [19]. These tasks were also presented using E-Prime software (version 2.0.10, Psychology Software Tools; scripts available at osf.io/m8r76 [32]).

### Procedure

Participants of the behavioral sample completed both the story-reading phase as well as the rating phase of the SNPT-R on a laptop or personal computer in a quiet environment, at the Faculty of Social and Behavioural Sciences, Leiden University (adult participants) or at a secondary school in the Netherlands (adolescent participants). After performing the SNPT-R, participants completed, depending on their age, the self-report version of the Liebowitz Social Anxiety Scale [33] or the Social Anxiety Scale for adolescents [34], and the Brief Fear of Negative Evaluation-R scale [35]. These results are not discussed in the present paper.

Participants of the imaging sample performed the story-reading phase of the SNPT-R in the MRI scanner, located at the Leiden University Medical Center (LUMC). Imaging data were collected during the story-reading phase using a Philips 3.0 T Achieva MRI scanner (Philips Medical Systems, Best, The Netherlands), equipped with a 32-channel SENSE (Sensitivity Encoding) head coil. During the two blocks of the story-reading phase, functional scans were acquired using T2* weighted echo-planar imaging (repetition time (TR) = 2200 ms, echo time (TE) = 30 ms, 38 axial slices, descending acquisition, 2.75 mm × 2.75 mm × 2.75 mm + 10% interslice gap, field of view (FOV) = 220 mm × 115 mm × 220 mm, 230 volumes/block). The first six volumes of these scans were dummy volumes and removed to allow for equilibration of T1 saturation effects. A 3D T1-weighted anatomical scan was acquired for within-subject registration purposes before the SNPT-R (TR = 9.8 ms, TE = 4.59 ms, flip angle = 8◦, 140 slices, 0.875 mm × 0.875 mm × 1.2 mm, FOV = 224 mm × 168 mm × 177.333 mm). The task was part of a larger scanning session including other fMRI tasks, a resting-state scan, and a diffusion tensor imaging (DTI) scan.

Following the scan-session, participants performed the rating phase of the SNPT-R on a laptop in a quiet room next to the MRI scanner.

### Data analysis

#### Behavioral ratings

Statistical analyses of the ratings of embarrassment and inappropriateness for the stories of the SNPT-R were performed using IBM SPSS Statistics for Windows (Version 23.0. Armonk, NY: IBM Corp.). Internal consistency of the task conditions (intentional, unintentional and neutral) was determined by calculating Cronbach’s α for the ratings of inappropriateness and embarrassment, and for the diference score (again both for inappropriateness and embarrassment) between the intentional and unintentional condition for each set of stories.

Repeated measures ANOVAs with condition (intentional, unintentional, neutral) as a within-subjects factor were used to investigate differences between task conditions. Furthermore, group (based on the version of the SNPT-R; group 1: boys < 18 years of age; group 2: girls < 18 years of age; group 3: men ≥ 18 years of age; group 4: women ≥ 18 years of age) was added as a between-subjects factor. The SPSS code for analysis of the behavioral data is available at osf.io/m8r76 [32]. For all analyses, significance level was set at p ≤ 0.05 and Greenhouse–Geisser correction was used when the assumption of sphericity was violated.

#### Imaging data

Analysis of fMRI data was performed using FEAT (FMRI Expert Analysis Tool; version 6.00) [36,37], (FSL, RRID:SCR_002823; scripts available at osf.io/m8r76 [32]). Prestatistics processing consisted of motion correction using MCFLIRT [38], non-brain removal using BET [39], spatial smoothing using a Gaussian kernel of full-width half-maximum (FWHM) 6.0 mm, grand-mean intensity normalization of the entire 4D dataset by a single scaling factor in order to enable higher-level analyses, and high-pass temporal filtering (Gaussian-weighted least-squares straight line fitting, with σ = 30.0 s). Functional scans of each participant were registered to the individual 3D T1-weighted anatomical scan using FLIRT [38,40] and subsequently registered to the Montreal Neurological Institute (MNI) T1-template brain (resolution 2 mm) using FNIRT nonlinear registration [41]. Next, event-related statistical analysis of the time-series was carried out in native space using FILM with local autocorrelation correction [42]. For each participant, four explanatory variables (EVs) with their temporal derivatives were included in the general linear model, representing the presentation of (1) a stem sentence, (2) a neutral ending sentence, (3) an unintentional norm violation ending and (4) an intentional norm violation ending. Onset of the EVs was determined using custom-written scripts in Matlab (Mathworks; code available at osf.io/m8r76 [32]). The stem EV had a duration of 3 s, the ending EVs had a duration of 6 s. EVs were convolved with a double gamma hemodynamic response function. In addition, nuisance regressors were included for time-points corresponding to motion outliers using the FSL motion outliers program (http://fsl.fmrib.ox.ac.uk/fsl/fslwiki/FSLMotionOutliers), which defined outlier time-points using the 75^th^ percentile plus 1.5 times the InterQuartileRange criterion. The mean number of excluded time-points for block 1 and 2 of the story-reading phase of the SNPT-R was 12.00 (range: 1–24 volumes) and 13.24 (range: 3–26 volumes), corresponding to respectively 5.2% and 5.8% of the volumes for each block.

Subsequently, four contrasts of interest were defined, following the contrasts described by Berthoz et al. [17]: (1) intentional norm violation endings > neutral endings; (2) unintentional norm violation endings > neutral endings; (3) intentional norm violation endings > unintentional norm violation endings; (4) unintentional norm violation endings > intentional norm violation endings. We verified whether the individual scans were registered correctly and confirmed that relative motion parameters did not exceed 2.5 mm. Subsequently, the individual contrast images of the two story-reading blocks of the SNPT-R were combined using a within-subject multi-session fixed-effects analysis and the resulting contrast images were submitted to higher-level mixed-effects group analyses using FMRIB’s Local Analysis of Mixed Effects (FLAME-1) [43–45]. We performed whole-brain analyses to investigate clusters related to the four contrasts of interest and tested clusters for significance using a height threshold of z > 2.3 and a cluster-corrected significance threshold of p < 0.05, using Gaussian random field theory [46]. In addition, we determined, in line with the analysis described by [17], common areas activated by the intentional and unintentional norm violations by applying a binary mask of the areas significantly activated by contrast 2 (unintentional norm violation endings > neutral endings) to contrast 1 (intentional norm violation endings > neutral endings), again using the above-mentioned statistical thresholds.

Furthermore, we investigated, following Berthoz and colleagues [18] who re-analysed the data of [17] to test the hypothesis that the amygdala is pivotal in processing one’s own intentional social norm transgressions, a hypothesis which was confirmed, activation within the left amygdala for the contrasts involving intentional norm violations. Therefore, we used a mask that was created in standard space (resolution 2 x 2 x 2 mm) using the Harvard-Oxford Subcortical Structural Atlas implemented in FSLView (version 3.2.0), which included voxels with a probability of at least 50% of belonging to the left amygdala. Again, a height threshold of z > 2.3 and a cluster-corrected significance threshold of p < 0.05 was used.

Unthresholded statistical maps have been uploaded to NeuroVault.org [47] and are available at http://neurovault.org/collections/QCZKVNWZ/ as well as at osf.io/m8r76 [32].

## Results

### Behavioral ratings

#### Task characteristics

The items of the SNPT-R for each condition were shown to have good internal consistency with respect to the ratings of both embarrassment (intentional: Cronbach’s α = 0.94; unintentional: Cronbach’s α = 0.90; neutral: Cronbach’s α = 0.73) and inappropriateness (intentional: Cronbach’s α = 0.85; unintentional: Cronbach’s α = 0.88; neutral: Cronbach’s α = 0.66). Furthermore, Cronbach’s α on the difference scores (intentional vs. unintentional) was high (embarrassment: Cronbach’s α = 0.90; inappropriateness: Cronbach’s α = 0.84), indicating that the stories were internally consistent with respect to the difference between the intentional and unintentional condition.

#### Differences between task conditions and effects of group (behavioral sample)

**Fig 2:**
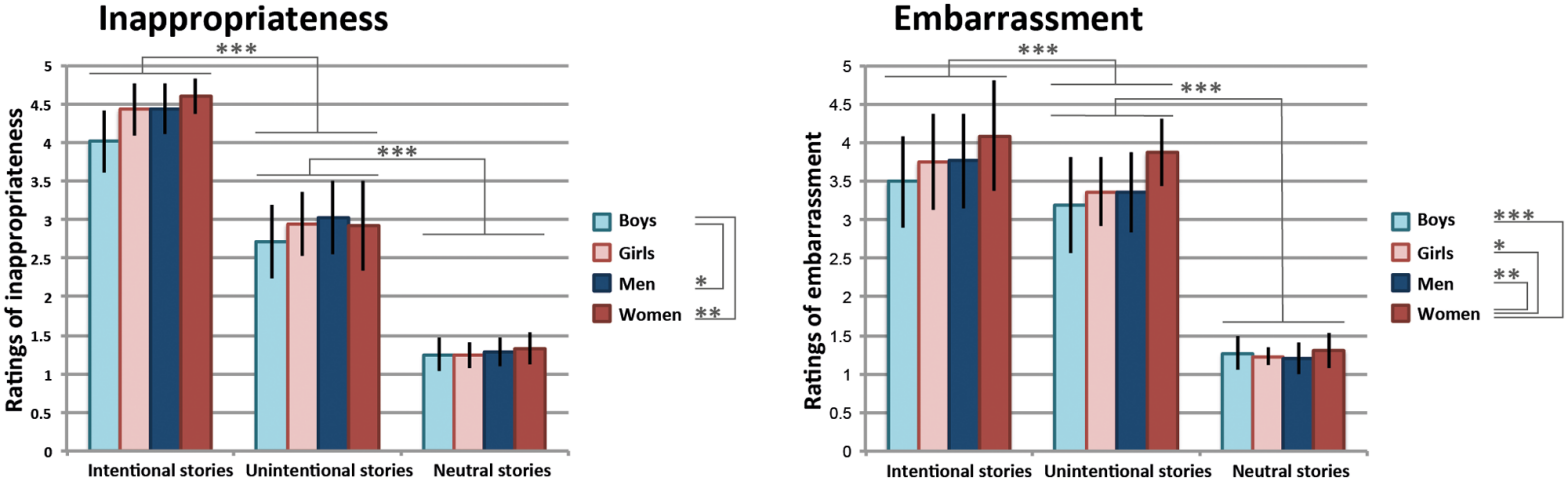
Behavioral ratings on the SNPT-R (*n* = 87, behavioral sample). Stories describing intentional social norm violations were rated as more inappropriate and more embarrassing when compared to stories on unintentional social norm violations, while unintentional stories were considered more inappropriate and more embarrassing in comparison to neutral stories. Boys rated the stories as less inappropriate when compared to men and women; women rated the stories as more embarrassing in comparison to the other groups. Data are presented as means ± SD. *: p ≤ 0.05; **: p ≤ 0.01; ***: p ≤ 0.001.

Ratings for the three task conditions of the SNPT-R (behavioral sample) are presented in Table 2 and Fig 2 (for ratings at individual and story level, we refer the reader to the S1 Dataset; original E-Prime output files and csv files are also available at osf.io/m8r76 [32]). Given the unequal sample sizes, we checked whether the variances were significantly different between the groups. This was not the case: for both the embarrassment and inappropriateness data, Box’s Test of Equality of Covariance Matrices was not significant (inappropriateness: Box’s M = 20.7, F(18, 10272.5) = 1.1, p = 0.38; embarrassment: Box’s M = 17.4, F(18, 10272.5) = 0.89, p = 0.59). Furthermore, Levene’s Test of Equality of Error Variance was not significant (inappropriateness intentional: F(3,83) = 2.14, p = 0.10; inappropriateness unintentional: F(3,83) = 1.01, p = 0.39; inappropriateness neutral: F(3,83) = 0.49, p = 0.69; embarrassment intentional: F(3,83) = 0.50, p = 0.69; embarrassment unintentional F(3,83) = 1.40, p = 0.25; embarrassment neutral: F(3,83) = 1.13, p = 0.34), indicating that the assumptions for interpreting the results of the repeated measures ANOVA are met.

**Table 2:** Ratings of inappropriateness and embarrassment for the SNPT-R—behavioral sample.

	Inappropriateness			Embarrassment		
	Intentional stories	Unintentional stories	Neutral stories	Intentional stories	Unintentional stories	Neutral stories
**Total sample (*n* = 87)**	**4.43 ± 0.36**	**2.93 ± 0.51**	**1.29 ± 0.20**	**3.83 ± 0.67**	**3.50 ± 0.56**	**1.25 ± 0.21**
Boys (*n* = 13)	4.01 ± 0.40	2.71 ± 0.48	1.25 ± 0.21	3.49 ± 0.59	3.19 ± 0.63	1.27 ± 0.22
Girls (*n* = 16)	4.43 ± 0.34	2.94 ± 0.41	1.24 ± 0.17	3.75 ± 0.62	3.36 ± 0.45	1.23 ± 0.12
Men (*n* = 29)	4.44 ± 0.33	3.03 ± 0.48	1.29 ± 0.19	3.76 ± 0.62	3.35 ± 0.52	1.20 ± 0.20
Women (*n* = 29)	4.60 ± 0.23	2.92 ± 0.58	1.33 ± 0.21	4.09 ± 0.72	3.87 ± 0.44	1.31 ± 0.23

Data are presented as means ± SD.

Repeated measures ANOVAs (condition x group) showed significant effects of condition on both the ratings of embarrassment (F(1.7,144.4) = 790.8, p < 0.001, η^2^ = 0.90) and inappropriateness (F(1.7,137.1) = 2018.5, p < 0.001, η^2^ = 0.96). In addition, there were significant effects of group on the ratings of embarrassment (F(3,83) = 7.02, p < 0.001, η^2^ = 0.20) and ratings of inappropriateness (F(3,83) = 3.9, p = 0.011, η^2^ = 0.12), as well as interaction effects between group and condition (embarrassment: F(5.2,144.4) = 2.5, p = 0.03, η^2^ = 0.009; inappropriateness: F(5.0,137.1) = 3.0, p = 0.01, η^2^ = 0.004) (Fig 2).

Post-hoc paired-samples t-tests showed that the mean ratings of inappropriateness were significantly higher for the intentional stories relative to the unintentional stories (t(86) = 27.7, p < 0.001, r = 0.95), while the unintentional stories were rated as more inappropriate compared to the neutral stories (t(86) = 34.0, p < 0.001, r = 0.96). A similar pattern was found for the ratings of embarrassment: participants rated the intentional stories as the most embarrassing (intentional > unintentional: t(86) = 4.6, p < 0.001, r = 0.44), and the unintentional stories as more embarrassing when compared to the neutral stories (t(86) = 40.3, p < 0.001, r = 0.97). Separate repeated measures ANOVAs for each group confirmed that the effect of condition was significant for all age- and gender specific versions of the task, both for inappropriateness and embarrassment (effect of condition on inappropriateness: boys: F(2,24) = 255.0, p < 0.001, η^2^ = 0.96; girls: F(2,30) = 627.8, p < 0.001, η^2^ = 0.98; men: F(2,56) = 709.8, p < 0.001, η^2^ = 0.96; women: F(1.4,39.3) = 845.8, p < 0.001, η^2^ = 0.97; effect of condition on embarrassment: boys: F(2,24) = 99.2, p < 0.001, η^2^ = 0.89; girls: F(2,30) = 146.9, p < 0.001, η^2^ = 0.91; men: F(2,56) = 356.0, p < 0.001, η^2^ = 0.93; women: F(1.5,42.3) = 351.8, p < 0.001, η^2^ = 0.93).

Post-hoc tests (corrected for multiple comparisons using Bonferroni correction) indicated that boys rated the stories as less inappropriate when compared to men (p = 0.03) and women (p = 0.01), while a follow-up oneway ANOVA showed that this effect was specific for the intentional condition (F(3,86) = 10.6, p < 0.001, η^2^ = 0.28), with significant differences between boys and the other groups (Bonferroni-corrected comparisons: boys < girls, p = 0.003; boys < men: p = 0.001; boys < women: p < 0.001). There were no group differences with respect to the ratings of inappropriateness for the unintentional (F(3,86) = 1.2, *ns*) and neutral stories (F(3,86) = 0.9, *ns*).

Women rated the stories overall as more embarrassing in comparison to boys (p = 0.001), girls (p = 0.03) and men (p = 0.003), and a follow-up oneway ANOVA indicated that this effect was present in the intentional (F(3,86) = 2.9, p = 0.04, η^2^ = 0.10; women > boys, p = 0.04) and the unintentional condition (F(3,86) = 8.2, p < 0.001, η^2^ = 0.23; women > boys: p = 0.001; women > girls: p = 0.009; women > men: p = 0.001; all comparisons Bonferroni-corrected for multiple comparisons). There were no differences between the groups with respect to the embarrassment-ratings of the neutral condition (F(3,86) = 1.7, *ns)*.

#### Differences between task conditions and effects of group (imaging sample)

Ratings for the three task conditions of the SNPT-R (imaging sample) are presented in Table 3 (for ratings at individual and story level, we refer the reader to S2 Dataset; original E-Prime output files and csv files are also available at osf.io/m8r76 [32]). Repeated measures ANOVAs replicated all significant effects found in the behavioral sample. That is, there was a significant effect of condition for both the ratings of embarrassment (F(2,38) = 216.1, p < 0.001, η^2^ = 0.91) and inappropriateness (F(1.5,28.2) = 271.0, p < 0.001, η^2^ = 0.92), with the highest ratings of embarrassment and inappropriateness for the intentional stories (embarrassment: intentional > unintentional: t(20) = 3.9, p = 0.001, r = 0.66; unintentional > neutral: t(20) = 17.3, p = 0.001, r = 0.97; inappropriateness: intentional > unintentional: t(20) = 17.9, p < 0.001, r = 0.97; unintentional > neutral: t(20) = 12.0, p < 0.001, r = 0.94). Furthermore, there were significant effects of group on the ratings of embarrassment and inappropriateness (embarrassment: F(1,19) = 5.8, p = 0.03, η^2^ = 0.23; inappropriateness: F(1,19) = 4.7, p = 0.04, η^2^ = 0.20), with higher ratings for women compared to men. In addition, results showed a significant interaction between condition and group on the ratings of inappropriateness (F(1.5, 28.2) = 4.4, p = 0.03, η^2^ = 0.01), while this interaction was significant at trend level for the ratings of embarrassment (F(2,38) = 3.0, p = 0.06, η^2^ = 0.01). Oneway ANOVAs indicated that women rated intentional social norm violations as more inappropriate relative to men (F(1,20) = 5.4, p = 0.03, η^2^ = 0.22), and unintentional social norm violations as both more inappropriate (F(1,20) = 5.7, p = 0.03, η^2^ = 0.23) and more embarrassing (F(1,20) = 7.6, p = 0.01, η^2^ = 0.29). The other comparisons were not significant (embarrassment intentional: F(1,20) = 3.3, *ns*; embarrassment neutral: F(1,20) = 0.25, *ns*; inappropriateness neutral: F(1,20) = 0.14, *ns*).

**Table 3:** Ratings of inappropriateness and embarrassment for the SNPT-R—imaging sample.

	Inappropriateness			Embarrassment		
	Intentional stories	Unintentional stories	Neutral stories	Intentional stories	Unintentional stories	Neutral stories
**Total sample (*n* = 21)**	**4.37 ± 0.49**	**3.11 ± 0.60**	**1.40 ± 0.32**	**4.00 ± 0.62**	**3.53 ± 0.60**	**1.33 ± 0.27**
Men (*n* = 6)	4.01 ± 0.71	2.66 ± 0.77	1.44 ± 0.57	3.64 ± 0.60	3.03 ± 0.72	1.28 ± 0.34
Women (*n* = 15)	4.51 ± 0.30	3.28 ± 0.44	1.38 ± 0.18	4.16 ± 0.59	3.73 ± 0.43	1.35 ± 0.24

Data are presented as means ± SD.

### Imaging data

#### Behavioral responses during story-reading phase

We verified whether participants engaged with the task during the story-reading phase by examining the behavioral responses of the participants (i.e. a button press during the presentation of the stem sentence). On average, participants responded to 96% of trials (number of missed responses / block of 39 trials (mean ± SD): 1.6 ± 1.8, range 0–8), indicating good task compliance.

#### Intentional norm violations versus neutral stories

**Fig 3:**
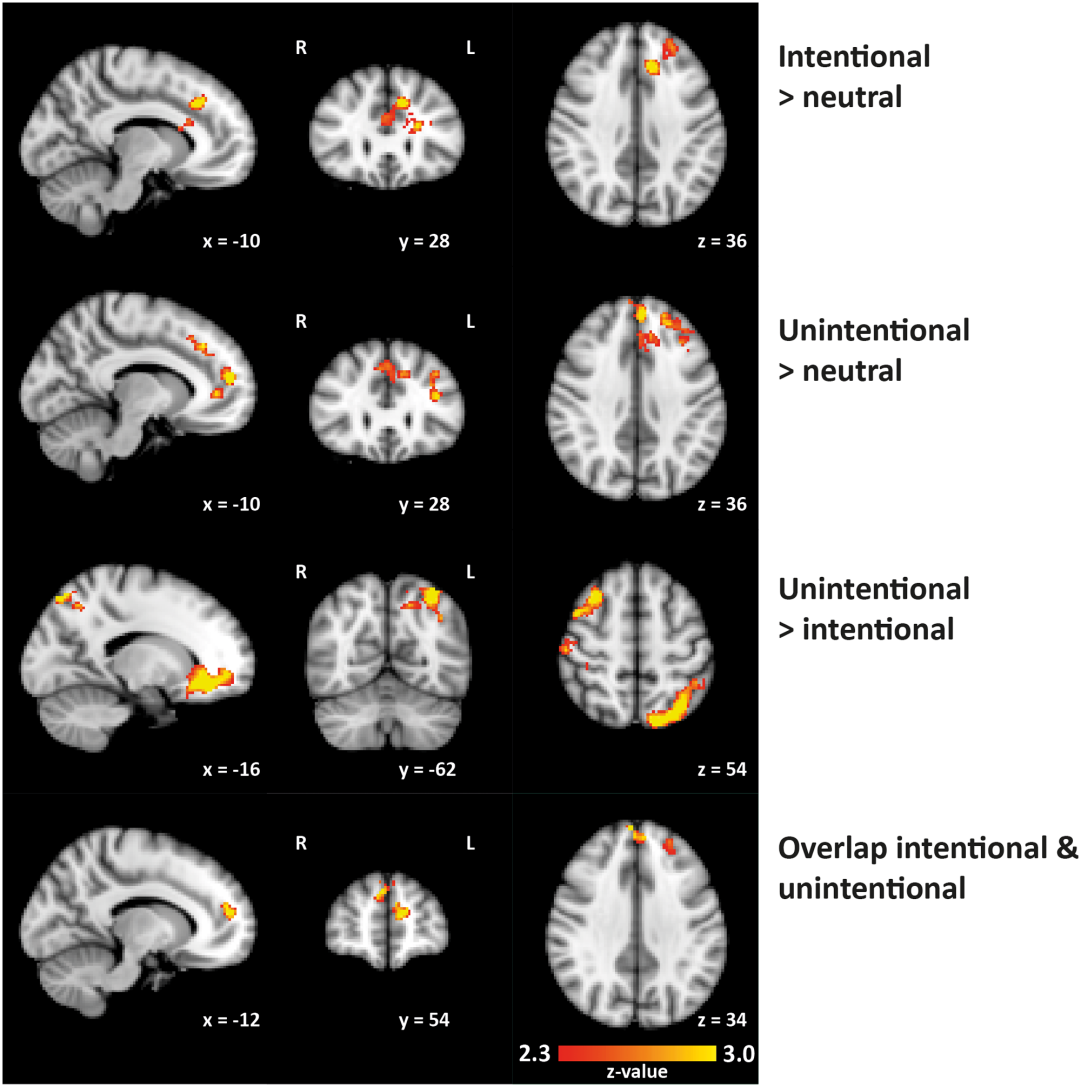
Significant activation clusters related to processing stories describing social norm violations. Clusters are superimposed on the template MNI_T1_152_2mm_brain (partial brain coverage; inferior parts of the frontal medial cortex and superior parts of the postcentral gyrus are not included). All images are displayed according to radiological convention: right in image is left in brain.

Reading stories describing intentional social norm violations evoked activation in a cluster encompassing the paracingulate gyrus, superior frontal gyrus and frontal pole, extending into the left inferior frontal gyrus, frontal operculum cortex and left caudate (p = 0.01; cluster-size 748 voxels; peak coordinate in MNI space: X = -10, Y = 28, Z = 36; peak z-value = 3.39), when compared to reading neutral stories (Table 4; Fig 3). Furthermore, significant activation was present in the left amygdala, revealed by a post-hoc analysis using a mask of the left amygdala (p = 0.033; cluster-size 21 voxels; peak coordinate in MNI space: X = -18, Y = -10, Z = -12; peak z-value = 3.15).

**Table 4:** Brain activity related to reading social stories describing intentional and unintentional norm violations versus neutral situations.

Cluster	Region	Z-score	Peak coordinates (MNI space)			Cluster size
			x	y	z	
**Intentional norm violations vs neutral stories**						
1	Left paracingulate gyrus / superior frontal gyrus	3.39	-10	28	36	748
	Left frontal pole	2.99	-26	40	40	
	Left frontal operculum cortex	2.87	-44	12	6	
	Left amygdala*	*3*.*15*	*-18*	*-10*	*-12*	*21*
**Unintentional norm violations vs neutral stories**						
1	Left paracingulate gyrus / superior frontal gyrus	3.99	-14	52	14	1604
	Left superior frontal gyrus	3.72	-4	46	38	
	Left middle frontal gyrus	3.46	-36	30	20	
	Right superior frontal gyrus	3.46	8	52	28	
	Left frontal pole	3.39	-20	42	32	
**Intentional versus unintentional norm violations**						
	*No significant clusters*					
**Unintentional versus intentional norm violations**						
1	Left orbitofrontal cortex	4.39	-26	36	-14	2179
	Left paracingulate gyrus	3.52	-10	48	-6	
	Right frontal medial cortex	3.52	2	52	-8	
	Subcallosal cortex	3.46	2	26	-8	
2	Right postcentral gyrus	3.42	38	-36	66	982
	Right middle frontal gyrus	3.30	32	20	54	
3	Left lateral occipital cortex	3.80	-34	-64	58	926
	Left superior parietal lobule	3.36	-36	-58	48	
**Overlap unintentional and intentional norm violations**						
1	Right superior frontal gyrus	3.43	4	56	34	167
	Left superior frontal gyrus	3.15	-6	50	36	
2	Left paracingulate gyrus	3.23	-12	52	16	150
	Left superior frontal gyrus	2.98	-6	54	22	
3	Left frontal pole	2.99	-26	40	40	98

*: post-hoc analysis using mask of left amygdala

#### Unintentional norm violations versus neutral stories

Reading stories describing unintentional social norm violations evoked activation in a cluster including the left superior frontal gyrus, left middle frontal gyrus, left frontal pole, left paracingulate gyrus and right superior frontal gyrus (p < 0.001; cluster-size 1604 voxels; peak coordinate in MNI space: X = -14, Y = 52, Z = 14; peak z-value = 3.99), when compared to reading neutral stories (Table 4; Fig 3).

#### Intentional versus unintentional norm violations

There were no clusters where reading the intentional norm violations evoked more activation when compared to reading the unintentional norm violations (using a height threshold of z > 2.3 and a cluster-corrected significance threshold of p < 0.05). Even when we restricted the analysis to the regions reported in [17], using a region of interest approach (spheres with a radius of 5 mm around the coordinates reported for the contrast intentional > unintentional transgressions) and applied a liberal threshold (p < 0.05, uncorrected), no significant activation was found. Furthermore, no activation was present in the left amygdala.

#### Unintentional versus intentional norm violations

Comparison of brain activation related to reading the unintentional norm violations versus intentional norm violations revealed three clusters (Table 4; Fig 3). The first cluster contained the left orbitofrontal cortex, left paracingulate gyrus and subcallosal cortex, and extended into the right frontal medial cortex (p < 0.001; cluster-size 2179 voxels; peak coordinate in MNI space: X = -26, Y = 36, Z = -14; peak z-value = 4.39). The second cluster encompassed the right postcentral gyrus and right middle frontal gyrus (p = 0.002; cluster-size 982 voxels; peak coordinate in MNI space: X = 38, Y = -36, Z = 66; peak z-value = 3.42), the third cluster was located in the left lateral occipital cortex and the left superior parietal lobule (p = 0.003; cluster-size 926 voxels; peak coordinate in MNI space: X = -34, Y = -64, Z = 58; peak z-value = 3.80).

#### Overlap between intentional and unintentional norm violations

In line with the work of Berthoz and colleagues [17], we also examined common areas activated by the intentional and unintentional norm violations. We created a binary mask of the significant activation cluster of contrast 2 (unintentional norm violation endings > neutral endings) and investigated activation related to contrast 1 (intentional norm violation endings > neutral endings) within this mask. We found three clusters of common activation (Table 4; Fig 3): a cluster encompassing left and right superior frontal gyrus (p = 0.02; cluster-size 167 voxels; peak coordinate in MNI space: X = 4, Y = 56, Z = 34; peak z-value = 3.43), a cluster in the left paracingulate gyrus extending into the left superior frontal gyrus (p = 0.02; cluster-size 150 voxels; peak coordinate in MNI space: X = -12, Y = 52, Z = 16; peak z-value = 3.23) and a cluster in the left frontal pole (p = 0.05; cluster-size 98 voxels; peak coordinate in MNI space: X = -26, Y = 40, Z = 40; peak z-value = 2.99).

## Discussion

In the present study, we investigated the behavioral and neural correlates of social norm processing in two independent samples, using a new instrument: the revised Social Norm Processing Task (SNPT-R). The SNPT-R, based on a task originally developed by Berthoz and colleagues [17,18] and used by [19], entails three conditions, allowing the investigation of the neural responses and behavioral ratings related to processing 1) stories describing intentional violations of social norms, 2) stories on unintentional violations of social norms, and 3) neutral social stories (Fig 1), in both adolescents and adults. We examined the behavioral ratings of the stories (concerning inappropriateness and embarrassment) in a sample of adolescents and adults (*n* = 87), and examined both the behavioral as well as the neural correlates of social norm processing using functional Magnetic Resonance Imaging (fMRI) in an independent sample of 21 adults. Our overall aim was to replicate the results from previous versions of the SNPT [17–19] and to describe the characteristics of the SNPT-R in detail, in order to enable the use of this paradigm in future studies involving both healthy participants and patient populations. Findings are discussed below.

### Ratings of embarrassment and inappropriateness: Dependent on type of story

In a large sample of adolescents and adults, we examined the ratings of inappropriateness and embarrassment concerning the three types of stories. Because all stories were written in second person (‘you’) and participants were asked to imagine themselves in the situation described, the ratings reflect how participants evaluate their own social norm transgressions. Results indicated a consistent effect of condition: participants rated the stories describing intentional social norm violations as the most inappropriate and the most embarrassing, while the unintentional social norm transgressions were rated more inappropriate and more embarrassing than the neutral stories (Table 2; Fig 2). These effects of condition were confirmed in the behavioral ratings by another, independent sample (*n* = 21) of adults (Table 3). Again, intentional social norm violations were rated as more inappropriate and more embarrassing than unintentional social norm violations. It is important to mention that we aimed to keep the actual outcomes of the intentional and unintentional social norm transgressions as far as possible the same. Thereby, these results indicate that participants consider their intention of importance for the evaluation of the transgression. The higher levels of inappropriateness for the intentional social norm violations indicate that participants are familiar with social conventions, while we hypothesize that the higher levels of embarrassment for the intentional social norm violations indicate that participants 1) realize that intentional actions decrease their personal reputation to a greater extent than unintentional actions [48], and 2) that they are aware that intentional social norm violations require more prosocial behavior (i.e. by communicating to others that they recognize and regret their misbehavior and that they will do better in the future, as defined by [49]) than unintentional social norm violations.

Our finding with respect to the pattern of inappropriateness ratings is in line with the results of Berthoz and colleagues, demonstrating that healthy males (*n* = 12) rated intentional norm violations as more inappropriate than unintentional norm violations [17]. However, the ratings of embarrassment reported here do not coincide with those described in Berthoz et al. [17], who found that mean embarrassment ratings were significantly higher for the unintentional social norm violations than for the intentional social norm violations. Nevertheless, our results seem to be in line with the behavioral ratings on embarrassment by healthy participants (*n* = 16) in the study by Blair et al. [19], showing slightly higher ratings of embarrassment for intentional than for unintentional social norm violations—although this study did not statistically test within-group differences between the task conditions. These discrepancies stress the need for replication studies.

It is important to note that the SNPT-R differs from previous versions of the paradigm [17–19] in the sense that the SNPT-R includes four age- and gender specific versions: for boys < 18 years of age, girls < 18 years of age, men ≥ 18 years of age and women ≥ 18 years of age. These versions were created in order to maximize the personal involvement of participants with the task, which was important because we aimed to investigate the behavioral and neural responses involved in evaluating one’s own actions (cf. [13]). We investigated whether the effect of condition on inappropriateness and embarrassment was present in all participant groups. Results showed that this was indeed the case, indicating that all four versions of the SNPT-R enable distinguishing intentional and unintentional social norm violations based on behavioral ratings of inappropriateness and embarrassment. We did find, however, differences between the groups: boys considered the stories as less inappropriate when compared to the adult groups (both men and women), while women reported higher levels of embarrassment when rating the stories (in comparison to all other groups; Fig 2). We hypothesize that these effects are due to gender differences and developmental changes in moral sensitivity [50–52], but future research is needed to examine this in detail.

### Processing stories on social norm violations: Overlapping and differential activation patterns for intentional and unintentional violations

Imaging results showed that reading stories describing social norm violations (both intentional and unintentional) evoked overlapping activation within the frontal pole, the paracingulate gyrus, and the superior frontal gyrus, relative to reading neutral social stories (Table 4; Fig 3). Furthermore, we observed activation within the middle frontal gyrus related to reading unintentional social norm violations when compared to reading neutral stories, while reading intentional social norm violations (in comparison to reading neutral stories) evoked activation within the frontal pole, paracingulate gyrus and frontal operculum cortex. In addition, reading stories on intentional social norm transgressions was related to activation in the left amygdala. When contrasting unintentional and intentional norm violations, differential activation was found within three clusters: a cluster encompassing the left orbitofrontal cortex, frontal medial cortex and subcallosal cortex, a cluster involving the right postcentral gyrus and right middle frontal gyrus, and an occipital-parietal cluster (Table 4; Fig 3). There were no clusters where reading the intentional norm violations evoked more activation in comparison to the unintentional norm violations.

These results are largely in line with the findings of Berthoz and colleagues [17] who investigated the neural systems underlying the processing of social norm transgressions in a sample of twelve healthy male participants; they reported activation in several regions in the medial prefrontal cortex in response to social norm violations, as well as in the orbitofrontal cortex, temporo-parietal regions and the basal temporal cortex. Furthermore, a re-analysis of the same dataset revealed enhanced activation in the left amygdala in response to intentional social norm violations, a finding that was replicated in the present study. In addition, our findings coincide with the results of neuroimaging studies considering brain activation related to thinking about the self and thinking about others, and of studies on moral reasoning—processes which are important in evaluating social norm violations [10,13–15,53–56]. More specifically, the paracingulate gyrus and superior frontal gyrus, activated by both intentional and unintentional social norm violations, have been implicated previously in mentalizing [54] and the experience of shame [15], embarrassment [14] and guilt [57], while activation within the frontal pole is associated with moral reasoning [10]. Furthermore, the ventral medial frontal cortex and orbitofrontal cortex, in this study activated by unintentional social norm violations, were found to be involved in self-related judgements [53], self-referential processing [56], moral emotions [55,58] and in evaluative processes of embarrassment [14]. Our results build upon these findings and provide more insight in the neural processes underlying dealing with one’s own social norm transgressions.

It should, however, be noted that we did not find significant clusters when contrasting intentional versus unintentional social norm violations, while Berthoz et al. [17] reported more pronounced activation in several prefrontal, temporal and parietal regions when investigating this contrast. This discrepancy is possibly due to differences in task parameters (the task employed by Berthoz and colleagues involved both personal and impersonal stories, as well as stories comprised of ‘unrelated words’ [17,18], while the SNPT-R only involved personal stories written in second-person) and the use of a more stringent statistical threshold in the present study. In addition, we cannot exclude the possibility that the participants’ initial reaction to the stories, while reading them in the MRI-scanner, differs from the reaction as reflected in the ratings after the scan session. These ratings indicated higher levels of embarrassment and inappropriateness for the stories on intentional social norm violations, but it is possible that unintentional transgressions evoked more arousal on the first time reading, which is reflected in increased activation levels in the brain. However, data to test this hypothesis are not available.

### Limitations and suggestions for future research

In line with previous work on the SNPT [17,19], we focused on the experience of embarrassment in relation to social norm violations. However, given the fact that social norm violations could also evoke other reactions, future studies could investigate how participants rate the stories with respect to the experience of other prosocial emotions like shame and guilt [3–5], as well as look into the potential positive outcomes of social norm transgressions for the transgressor [59].

A limitation of the present study is the relatively small sample size of the adolescent sample (13 boys and 16 girls). However, the distribution of the variances was not significantly different between the groups and the effect of condition on behavioral ratings was robustly present in all samples (all p < 0.001, both for ratings of inappropriateness and embarrassment), so we feel our data provide substantial support for the usefulness of the SNPT-R in these populations. Another shortcoming is the fact that we did not acquire imaging data in the adolescent sample. As a result, we were not able to investigate developmental changes in brain activation related to social norm processing. Given the fact that adolescence is a time period characterized by influential changes in social-affective and social cognitive abilities [60,61], it could be hypothesized that reading one’s own social norm violations evokes differential activation patterns in adolescents in comparison to adults. Future studies, in line with the behavioral study by Lahat and colleagues [62], could investigate this topic.

Furthermore, based on the results of Blair et al. [19], showing aberrant behavioral and neural responses to social norm violations in patients with SAD, and given the fact that social anxiety symptoms are present at a continuum, ranging from a total lack of symptoms to normal levels of social anxiety or even mild social fears, in the normal population [63], future studies could investigate the relation between self-report measures of social anxiety and behavioral ratings of social norm violations in healthy participants. In addition, we suggest that the SNPT-R could be used to investigate the behavioral and neural correlates of social norm processing in other patient populations in which disturbances of social behavior are present, for example in patients with frontal brain lesions, patients with frontotemporal dementia and patients with personality disorders. Using the SNPT-R across disorders is in line with the Research Domain Criteria project (RDoC), which proposes a framework for conducting research in which core symptoms (in this case: disturbances in social behavior) are studied at different levels and across diagnostic classifications of disorders, in order to gain more insight in the mechanisms underlying normal and abnormal behavior [64].

## Conclusions

To conclude, the data presented here provide support for the use of the SNPT-R to investigate the behavioral and neural substrates of social norm processing. Intentional social norm violations were rated as more inappropriate and more embarrassing when compared to unintentional social norm violations, while reading stories describing these violations evoked activation within the frontal pole, the paracingulate gyrus and the superior frontal gyrus. Furthermore, processing unintentional social norm violations was associated with activation in, among others, the orbitofrontal cortex, middle frontal gyrus and superior parietal lobule, while reading intentional social norm violations was related to activation in the left amygdala. These regions have been previously implicated in thinking about one’s self, thinking about others and moral reasoning. These findings indicate that the SNPT-R could serve as a useful paradigm for examining social norm processing, both at the behavioral and neural level.

## Supporting information

S1 Dataset Ratings on SNPT-R—Behavioral sample (*n* = 87). (CSV)

S2 Dataset Ratings on SNPT-R—Imaging sample (*n* = 21). (CSV)

S1 Table Full list of SNPT-R stories. (CSV)

S1 Text Sensitivity analysis. (DOCX)
