# Supplementary material for: How embarrassing! The behavioral and neural correlates of processing social norm violations
Source: PLoS One. 2017 Apr 25;12(4):e0176326. doi: 10.1371/journal.pone.0176326 (PMC5404760; doi:10.1371/journal.pone.0176326)
Supplement: S1 Text — (DOCX) [file pone.0176326.s004.docx]

**Supporting information – S1 Text**

**Janna Marie Bas-Hoogendam^1,2,3^ * , Henk van Steenbergen^1,3^, Tanja Kreuk^1^, Nic J.A. van der Wee^2,3^, P. Michiel Westenberg^1,3^; How embarrassing! The behavioral and neural correlates of processing social norm violations**

**Sensitivity analysis with respect to outcome of the intentional vs. unintentional condition**

We aimed to keep the actual outcome of the action described in the *intentional* condition the same as the outcome of the action described in the *unintentional* condition. However, as the editor pointed out in his comments on a previous version of this manuscript, the phrasing of the stories differs between the intentional and unintentional condition. In some stories, we only varied words like ‘purposefully’ and ‘by accident’, in other stories we used a different verb that in itself explained whether the action was intentional or unintentional. We chose to do so to make the paradigm more lively (we thought that only varying the words ‘purposefully’ and ‘by accident’ would make the task monotonous), and more realistic. However, these differences in phrasing could have induced differences in how participants considered the outcomes of the actions, which could have subsequently influenced their ratings of inappropriateness and embarrassment.

We performed a sensitivity analysis to investigate whether these phrasing-differences between the stories could have systematically influenced our results. First, we determined for each story whether the outcome of the intentional action could be considered different (more severe) relative to the effect of the action described in the unintentional condition. This seems to be the case for 10 stories (Table 1, Supporting information – S2 Text and table - page 3); for these stories, it is uncertain whether research participants consider the effects of the intentional and unintentional condition the same.

Second, to investigate the extent to which these stories might have influenced the embarrassment and inappropriateness ratings, we performed a sensitivity-analysis by excluding these stories. Repeated-measures ANOVAs (condition x group) on the remaining 16 stories showed the same effects as reported for the full set of stories: we found significant effects of condition on both the ratings of embarrassment (F(1.7,143.8) = 643.44, p < 0.001) and inappropriateness (F(1.8,150.8) = 1740.5, p < 0.001). Post-hoc paired-samples t-tests confirmed that the mean ratings of inappropriateness were significantly higher for the intentional stories relative to the unintentional stories (t(86) = 25.5, p < 0.001), while the unintentional stories were rated as more inappropriate compared to the neutral stories (t(86) = 33.0, p < 0.001). Furthermore, intentional stories were rated as more embarrassing than the unintentional stories (t(86)= 2.7, p = 0.007), while unintentional stories were more embarrassing relative to neutral stories (t(86) = 38.4, p < 0.001).

In our opinion, these findings indicate that the overall effect that we have reported in the manuscript does not depend on the stories in which the outcome of the intentional and the unintentional action could be considered to be different.

**Table 1 Comparison of outcome of intentional vs. unintentional condition**

| **Story** | **Intentional** | **Unintentional** | **Outcome same severity?** |
| --- | --- | --- | --- |
| 1 | You see the long queue, and push in at the front of the line | You don't see the long queue, and go and stand at the front | Questionable |
| 2 | You see a woman sunbathing and kick her leg | You don't notice a woman sunbathing and accidentally trip over her leg | Questionable |
| 3 | You walk over to a friend and throw the coke on him | You stumble and spill coke on a friend | Questionable |
| 4 | You throw the airplane at your teacher and the airplane hits his head | You throw the airplane at the open window, but the airplane hits your teacher on the head | Yes |
| 5 | You see that it's occupied but you open the door | You don't see that it's occupied and you open the door | Questionable |
| 6 | You dislike it and spit it out | You take a bite of the cookie, choke, and spit it out | Yes |
| 7 | You throw your drink over another guest | You trip, and spill your drink over another guest | Questionable |
| 8 | You see a woman below and spit on her head | You spit and hit a woman by accident | Yes |
| 9 | You do not like the food and spit it out | You feel unwell and vomit over the table | Questionable |
| 10 | You don't want to hold it in and pee on the floor in front of everyone | You can't hold it in and wet yourself in front of everyone | Questionable |
| 11 | You undress in front of everyone in order to change clothes | You undress, but suddenly classmates walk in on you | Yes |
| 12 | You see your teacher and burp in front of him | You don't see your teacher, and burp in front of him | Yes |
| 13 | You notice the sign on the wall but go into the women's toilet anyway | You don't see the sign on the wall and go into the women’s toilet | Yes |
| 14 | You dive in the water and take off your swimming trunks | You dive in the water and your swimming trunks fall off | Yes |
| 15 | You decide to fart loudly | You accidentally fart loudly | Yes |
| 16 | You purposefully kick the ball in your own team's goal | You accidentally kick the ball in your own team’s goal | Yes |
| 17 | You wipe your nose on the table cloth | Your nose drips by accident on the table cloth | Questionable |
| 18 | You use salt instead of sugar as a joke | You use salt instead of sugar without realizing | Yes |
| 19 | You decide not to turn up | You forget to go | Yes |
| 20 | You decide not to feed the hamster, and it dies | You forget to feed the hamster, and it dies | Yes |
| 21 | You purposefully dye it blue | You mess up and his hair becomes blue | Yes |
| 22 | You feel bored with the conversation and hang up without saying goodbye | The telephone slips out of your hand and the connection is lost | Yes |
| 23 | You decide to sleep and lean on your neighbour's shoulder | You fall asleep, slumped against your neighbour's shoulder | Questionable |
| 24 | You see your teacher coming and you slam the door in his face | You don't see your teacher coming and you shut the door right in front of him | Questionable |
| 25 | You sneeze in your classmate's face for a laugh | All of a sudden you have to sneeze and you sneeze in a classmate's face | Yes |
| 26 | You see the dog defecating on the street but you keep walking | You don't see the dog defecating on the street and you keep walking | Yes |
